# Is body mass index still a good tool for obesity evaluation?

**DOI:** 10.1590/2359-3997000000232

**Published:** 2016-11-01

**Authors:** Erika Bezerra Parente

**Affiliations:** 1 Departamento de Medicina Faculdade de Ciências Médicas Santa Casa de São Paulo São Paulo SP Brasil Disciplina de Endocrinologia, Departamento de Medicina, Faculdade de Ciências Médicas da Santa Casa de São Paulo (FCMSCSP), São Paulo, SP, Brasil

Obesity has been increasing worldwide during the last decades (1,2). According to the World Health Organization (WHO 2014), 17.3% of Brazilians are obese (3). The diagnosis of obesity has huge consequences as this disease is associated with several comorbidities like diabetes, cancer, cardiovascular diseases and also increased mortality (4). Considering the pandemic of obesity, there is a need of a simple, reliable and low-cost tool for obesity evaluation.

The definition of obesity is based on the percentage of fat mass excess related to the total body weight (5). The body fat changes along aging and gender. Mean percentage of body fat may ranges from 22.9% at age 16-19 years to 30.9% at age 60-79 years in males, while mean percentage of body fat may ranges from 32.0% at age 8-11 years to 42.4% at age 60-79 years, in females (6). Therefore, how to perform this specific diagnosis of body fat excess?

Skinfold measurement is globally used for obesity diagnosis since it is quite simple and inexpensive method. The correlation of skinfolds to dual-energy-X-ray (DXA), the gold-standard, is better in non-obese people as the first method underestimates fat mass in obese ones (7).

The bioelectrical impedance analysis (BIA) is another useful method to calculate percentage of body fat and has an advantage over skinfold as it can estimate trunk fat. It is not so expensive and shows a good correlation to DXA (7).

Another recognized and validated tool is the whole-body air displacement plethysmography (ADP). Unfortunately, it is expensive and not available in many centers. Compared to DXA, ADP can overestimate body fat percentage in thinner people and underestimate body fat percentage in heavier ones (8).

The most accurate technique is the analysis of body composition by DXA, however it is also expensive and the patient is exposed to radiation. Even though it is an accurate method, the estimation of fat and lean mass by DXA software depends on levels of hydration, potassium content or tissue density (9).

Computed tomography (CT) and magnetic resonance imaging (MRI) may be useful to evaluate visceral fat or intramyocellular fat, respectively. Nevertheless they are expensive and not practical (10,11).

And what about body mass index (BMI)? In this issue of *Archives of Endocrinology and Metabolism *(AE&M), two studies addressed the issue of efficacy of BMI for obesity diagnosis and hyperglycemia screening.

BMI has limitations regarding the ability to discriminate fat mass from lean mass, which can drives to obesity over diagnosis in well trained people with high percentage of lean mass. Porto and cols. (12) studied 3,822 military firefighters divided in groups according to abdominal strength by sit-up test, cardiorespiratory fitness and age, since the percentage of body fat changes along aging and fitness. They found a similar prevalence of obesity estimated by BMI (13.3%) compared to percentage of body fat (%BF) (15.9%) measured by skinfold method. They verified an agreement of 85.8% between BMI and skinfold, although BMI underestimated the prevalence of obesity with high specificity (≥ 81.2%) and a low sensitivity (≤ 67.0) in all subgroups. They also found that a BMI over 30 kg/m^2^ was highly specific to exclude obesity; however BMI misclassification occurred on intermediate BMI (27.0 to 30.0 kg/m^2^). A limitation of this trial was the use of skinfold as the reference method for %BF instead of DXA. Skinfold have limitations like poor reproducibility and variation among different populations (8); nevertheless the authors were careful to use Brazilian references. Probably would be better to have DXA as the reference for body composition; however its high cost is the main barrier to be used in a large sample size.

Another BMI limitation is its lack of ability to identify visceral fat which is relevant to metabolic diseases and cardiovascular risk. In this *AE&M *issue, Quadros and cols. (13) studied 1,139 schoolchildren aged from six to seventeen years in order to evaluate the ability of BMI, waist circumference (WC) and waist-to-height ratio (WHR) to discriminate hyperglycemia. Hyperglycemia prevalence was 6.6% and it was more commonly present in young people with excess of weight, high WC and high WHR. The accuracies to discriminate hyperglycemia were significant, but low, for the individual (BMI = 0.56; WC = 0.53; WHR = 0.55) and combined indicators (BMI + WC = 0.55; BMI + WHR = 0.55). In addition, it was shown that adding WC and WHR measurement did increase the accuracy of BMI to diagnosis hyperglycemia in this pediatric population. Albeit the correlation between BMI and metabolic diseases, WC and cardiovascular risk is well established in adult population, these relationships in children and adolescents are still controversial. Other authors as Kuba and cols. (14), who studied children from six to ten years old, verified different results: significant correlations between WHR and BMI z score with cardio metabolic risk markers. Differences among ethnics, age and fat distribution may change the correlation between anthropometric indicators and metabolic disease. These variables could be more important among children and adolescents as BMI, WC and fat distribution change along growth. Quadros and cols. (13) did not demonstrate advantage to add WC and WHR to BMI for hyperglycemia screen, however they performed only one measurement of fasting glucose and this can be a bias for under diagnosis. Although anthropometric indicators correlation to metabolic diseases in children and adolescents is still in discussion, they should continue be used because of their simplicity, low cost and noninvasive method.

Therefore, is body mass index still a good tool for obesity evaluation? The answer is: yes, it is. Albeit BMI does not define fat distribution, it is simple, reliable, with low cost and a good correlation with metabolic disease in adults; even though with some limitations in the pediatric population.
